# Large-Scale Morphological Network Efficiency of Human Brain: Cognitive Intelligence and Emotional Intelligence

**DOI:** 10.3389/fnagi.2021.605158

**Published:** 2021-02-24

**Authors:** Chunlin Li, Kaini Qiao, Yan Mu, Lili Jiang

**Affiliations:** ^1^CAS Key Laboratory of Behavioral Science, Institute of Psychology, Beijing, China; ^2^Department of Psychology, University of Chinese Academy of Sciences, Beijing, China

**Keywords:** MRI, morphological network, network efficiency, intelligence, aging

## Abstract

Network efficiency characterizes how information flows within a network, and it has been used to study the neural basis of cognitive intelligence in adolescence, young adults, and elderly adults, in terms of the white matter in the human brain and functional connectivity networks. However, there were few studies investigating whether the human brain at different ages exhibited different underpins of cognitive and emotional intelligence (EI) from young adults to the middle-aged group, especially in terms of the morphological similarity networks in the human brain. In this study, we used 65 datasets (aging 18–64), including sMRI and behavioral measurements, to study the associations of network efficiency with cognitive intelligence and EI in young adults and the middle-aged group. We proposed a new method of defining the human brain morphological networks using the morphological distribution similarity (including cortical volume, surface area, and thickness). Our results showed inverted age × network efficiency interactions in the relationship of surface-area network efficiency with cognitive intelligence and EI: a negative age × global efficiency (nodal efficiency) interaction in cognitive intelligence, while a positive age × global efficiency (nodal efficiency) interaction in EI. In summary, this study not only proposed a new method of morphological similarity network but also emphasized the developmental effects on the brain mechanisms of intelligence from young adult to middle-aged groups and may promote mental health study on the middle-aged group in the future.

## Introduction

Cognitive intelligence and emotional intelligence (EI) were generally regarded as different aspects of the human abilities (Gardner, 1987; Goleman, 1995), and they complemented each other in an additive manner (Van Rooy and Viswesvaran, 2004; Cote and Miners, 2006). Also, it was well-known that they were both related to life outcomes, including academic accomplishment, work performance, and longevity (Mayer et al., 2004; Petrides et al., 2004; Deary, 2008, 2012). As we grew older, cognitive intelligence and EI exhibited distinct trajectories of development (Williams et al., 2006, 2008; Giedd, 2008). Although not fully understood, different age groups showed differences in these behaviors that were both accompanied by the human brain morphological and functional changes (Schnack et al., 2015; Szymkowicz et al., 2016; Xia et al., 2019). In more detail, cognitive intelligence, such as memory and processing speed, showed a rapid increase during childhood and adolescence, which peaked around 20s to 30s and then gradually declined throughout the later part of the life span, particularly after 50 years of age (Salthouse, 2009, 2012, 2017; Singh-Manoux et al., 2012). Contrary to the negative effects of aging on cognitive intelligence, EI was relatively well-preserved or even improved during aging, which was supported by the evidence that older age was associated with a greater positivity bias, better emotional control, and better emotional stability (Gross et al., 1997; Lachman and Bertrand, 2001; Charles et al., 2003; Phillips and Allen, 2004).

Previous studies have reported that both anatomical (white matter [WM] tractography) and functional network efficiencies were related to cognitive intelligence (Li et al., 2009; van den Heuvel et al., 2009; Langer et al., 2012) and EI (Smith et al., 2018b; Dzafic et al., 2019) in adolescence, adults, and elderly groups. However, most of them focused on one age group, and no study investigated whether, and how, the associations between the human brain network efficiency and intelligence changed from young adults to the middle-aged group. In contrast, researchers found a developmental shift from a predominantly negative correlation between cognitive intelligence and cortical thickness in early childhood to a positive correlation in late childhood and adulthood (Shaw et al., 2006; Schnack et al., 2015), and the positive correlation continued in people older than 60 (Naumczyk et al., 2018). These findings of different brain mechanisms for different age groups provided some foresights to better understand the neural mechanism from developmental perspective. Also, the brain mechanisms linked to both the decline of cognitive intelligence and the increase or maintenance of EI remains to be answered. Studies using two different age groups (including young adults and the middle-aged group) and two different behavioral measurements (including cognitive intelligence and EI) would provide a unique perspective to understand the neural mechanisms of intelligence and aging.

A single measurement of the human brain morphology only captured one profile of the complexity of neurobiological changes associated with aging, and the multiple measurements of the human brain characteristics have been proved to better explain individual differences in intelligence (Rodrigue and Kennedy, 2011; Kievit et al., 2012; Ritchie et al., 2015). Moreover, the human brain was organized as an efficient network composed of, spatially distributed but functionally linked, the brain regions (Bullmore and Sporns, 2009; Vogel et al., 2010). Recent studies turned the focus from investigating the structural underpins of cognitive intelligence and EI using only one measurement of the brain characteristics to explore the brain mechanisms of intelligence using morphological networks (Li et al., 2009; Angel Pineda-Pardo et al., 2016). The existing methods of building the human brain morphological networks are WM tractography (diffusion-weighted tractography; Li et al., 2009; Koenis et al., 2018) and the structural covariance network (Mechelli et al., 2005; Alexander-Bloch et al., 2013). WM is substantially the bundle for information transfer composed of myelinated axons and very few neurons. In contrast to Diffusion Tensor Imaging (DTI), which provides a direct measurement of structural connectivity in the human brain, wher a long-range structural connectivity could not be reliably quantified (Jeurissen et al., 2019), the measurement of gray matter (GM; composed mainly of cell bodies) allowed the detections of both short-range and long-range structural connectivity (Alexander-Bloch et al., 2013). In addition, the data analysis of DTI was largely affected by head motion and may involve a large number of false-positive connections (Thomas et al., 2014; Maier-Hein et al., 2017). The structural covariance network of GM could only yield one correlation matrix for a group of subjects (Alexander-Bloch et al., 2013), which only represented the characteristics of the population and did not reveal the individual differences. Besides the two methods of constructing morphology network, there was also a new method constructing interregion similarity network for a single individual from 10 brain properties (Seidlitz et al., 2018; Morgan et al., 2019). Goulas et al. (2017) reported that the cytoarchitectonically similar areas of the brain region were more likely to be axonally connected to each other, which might result in the synchronization of the brain regions when facing a task or a joint action of a cognitive activity. Thus, compared with WM connection network and the structural covariance network, the morphological similarity could measure the connectivity between the brain regions in a single individual and might be the most accurate and rational method to reflect the information transfer between them. The similarity network using 10 brain properties from MRI and DTI data should be more reliable but may contain more limitations because of the limitation of every single morphological measurement. Therefore, combined with the graph theory, we proposed a new method of constructing a large-scale morphology network based on the distributions of cortical surface characteristics from a structural MRI (sMRI) scan (cortical volume, surface area, and thickness) to study the associations of the morphological network efficiency with cognitive intelligence and EI.

Here, we recruited 67 healthy participants (aging 18–64), who finished sMRI scanning, followed by the assessment of cognitive intelligence and EI. Additionally, we used the morphological similarity network of cortical volume (surface area and thickness) to explore the associations between network efficiency and intelligence, including cognitive and emotional perspectives at different ages. The graph theory was applied to explore the information processing in the large-scale morphological network of the human brain. The global efficiency corresponds to long-distance interactions and reflects the information integration over the whole network; the nodal efficiency reflects the information transfer ability of the parcel (node), whereas the local efficiency reflects the specialization of a single node within the network (Latora and Marchiori, 2001; Bullmore and Sporns, 2012). We attempted to answer the following questions: Were cognitive intelligence and EI related to distinct brain organization patterns? Were there developmental effects on the correlations between the brain network efficiency and intelligence?

## Materials and Methods

### Participants

Participants were recruited from local community by advertisements. The initial sample included 67 participants (32 males and 35 females; mean age = 32.79 ± 13.11; ranged from 18.59 to 64.30). All participants were invited for a detailed mental health interview using the Mini-International Neuro-Psychiatric Interview, and people with a history of major neuropsychiatric illness, head injury, alcohol, or drug abuse were excluded from the study. We also excluded people with MRI contraindications, including people with implants, pacemakers, brain surgery, current pregnancy, and very recent tattoos. In addition to the MRI scanning, the participants were also assessed with Wechsler Adult Intelligence Scale (WAIS; 4th edition, Chinese) and Schutte Self-Report Emotional Intelligence Scale (SSEIS; Chinese). The final sample included 65 participants. Participants who were absent from the MRI scanning (*n* = 1) or did not pass the mental health interview (*n* = 1) were excluded. The institutional review board of Institute of Psychology Chinese Academy of Sciences approved this study, and written informed consent was obtained from the individual participant prior to the data acquisition.

### Behavior Measures

#### Cognitive Intelligence

The WAIS, 4th edition (Chinese), was applied to measure cognitive intelligence (Wang et al., 2013). In the 4th edition, the full-scale intelligence quotient (FSIQ) is a composite score obtained from 10 subtests measuring two components of cognitive ability: the general ability index (GAI), which is the FSIQ of previous versions, and the cognitive proficiency index (CPI), which was proposed with the development of cognitive psychology. The GAI is comprised of two subsets: the verbal comprehension index (VCI, estimated by the subtest as follows: vocabulary, similarities, and information) and the perceptual reasoning index (PRI, estimated by the subtest as follows: block design, visual puzzles, and matrix reasoning); and the CPI is comprised of two subsets: the working memory index (WMI, estimated by the subtest as follows: arithmetic and digital span) and the processing speed index (PSI, estimated by the subtest as follows: coding and symbol search). The raw scores of the 10 subtests and the standardized scores were recorded for the FSIQ computation. We combined both the raw scores of 10 subsets and standardized scores in final statistical analysis.

#### Emotional Intelligence

The SSEIS was applied to measure EI. It is a valid assessment developed by Schutte et al. (1998) based on the original model of EI of Salovey and Mayer (1990). The Chinese version has a high reliability and validity, and it consists of 33 items, *via* a five-point Likert scale for all items, to measure four dimensions as follows: emotion perception, emotion management of the self, emotion management of others, and emotion utilization (Wang, 2002). Participants were asked to response on which “1” represented “not true of me” and “5” represented “very true of me.” The Cronbach's α in the present study was 0.903. We aimed to examine the associations of the human brain morphological network efficiency with EI total score and four subscale scores.

### Imaging Acquisition

All MRI images were collected on a Discovery MR750 3.0-T scanner (GE Healthcare) at Institute of Psychology Chinese Academy of Sciences. The participants completed a T1-weighted structural MRI scan (eyes closed) with a magnetization-prepared rapid gradient-echo (MPRAGE) sequence with the following parameters: repetition time (TR) = 6.652 ms, echo time (TE) = 2.928 ms, inversion time (T1) = 450 ms, flip angle (FA) = 12°, field of view = 256 mm × 256 mm, acquisition matrix = 256 × 256, slice thickness = 1.0 mm, 192 sagittal slices, and voxel size = 1 mm × 1 mm × 1 mm.

### Imaging Data Preprocessing

All the images were preprocessed by the Connectome Computation System (CCS), which was formulated by our lab using fMRI Software Library (FSL), Analysis of Functional Neuroimages (AFNI), and FreeSurfer (Xu T. et al., 2015). It focuses on the surface-based analysis compared to other resting state fMRI data analysis pipelines, and detailed descriptions of the system could be found in our previous publications (Jiang et al., 2015). The preprocessing comprised structural image preprocessing and functional image preprocessing. The structural image preprocessing included noise removal, brain extraction using the volBrain Automated MRI Brain Volumetry System (http://volbrain.upv.es; Manjon and Coupe, 2016), intensity inhomogeneity correction, segmentation of cerebrospinal fluid (CSF), WM, and GM, construction of the GM-WM (white surface) and GM-CSF interface (pial surface), and spatial registration by matching the cortical folding patterns across participants by recon-all in FreeSurfer and Gaussian spatial smoothing (FWHM = 6 mm). Finally, the 3D structure images were projected onto the *fsaverage5* standard cortical surface with 10,242 vertices per hemisphere.

### Quality Control

Quality control is very important for solid data analysis. For structural MRI, our quality control procedure (QCP) was as follows: (1) we performed visual inspection on all the original images and excluded participants with obvious structural brain abnormalities and significant motor artifacts during the scan; (2) CCS provides screenshots of the brain tissue segmentation as well as screenshots of pial and white surface reconstruction. We visually checked the screenshots, and participants with bad brain tissue segmentation and surface reconstruction were excluded from the subsequent analysis. All the participants passed the quality control.

The final sample included 65 participants. There was one participant who only underwent the EI test but did not undergo the cognitive intelligence test. Therefore, we had 64 participants for the cognitive intelligence analysis and 65 participants for the EI analysis. For further detailed visualization, the whole group was divided into two age groups: young adults and the middle-aged group. The descriptive information of two groups is shown in Table 1.

**Table 1:** Descriptive statistics of young adults and middle-aged group.

	***N***	**Sex** **(male: female)**	**Age (year)**		**Education (year)**	
			**Mean ± SD**	**Range**	**Mean ± SD**	**Range**
Young adults	34	17:17	26.45 ± 4.68	18.59–32.84	16.47 ± 2.61	9–22
Middle-aged group	31	15:16	49.17 ± 7.97	35.92–64.30	14.16 ± 3.37	8–22
Whole group	65	32:33	37.29 ± 13.11	18.59–64.30	15.37 ± 3.19	8–22

### Morphological Network

Using a large-scale brain network, parcellation developed by Yeo et al. (2011), which subdivided the entire cortical surface into 51 spatially connected parcels based on resting-state functional connectivity, we computed the total cortical volume and the total cortical surface area of each parcel by summing the volume or area of all vertices belonging to that parcel. Mean thickness was calculated by averaging the thickness of all the vertices within each parcel. We excluded the parcels whose vertex number was <50, and finally 32 parcels were reserved for the final group analysis, expanding across all the Yeo-7 networks: visual network, sensory motor network, dorsal attention network, ventral attention network, limbic network, frontoparietal network, and default mode network (see Table 2).

**Table 2:** The vertex number of reserved 32 brain regions.

**Brain region(lh)**	**Vertex number**	**Brain region(rh)**	**Vertex number**
Vis(lh)	1213	Vis(rh)	1266
SomMot(lh)	1590	SomMot(rh)	1612
DorsAttn_Post(lh)	627	DorsAttn_Post(rh)	614
DorsAttn_FEF(lh)	97	DorsAttn_FEF(rh)	98
SalVentAttn_ParOper(lh)	130	DorsAttn_PrCv(rh)	50
SalVentAttn_FrOper(lh)	331	SalVentAttn_TempOccPar(rh)	208
SalVentAttn_Med(lh)	216	SalVentAttn_FrOper(rh)	313
Limbic_OFC(lh)	213	SalVentAttn_Med(rh)	242
Limbic_TempPole(lh)	331	Limbic_OFC(rh)	237
Cont_Par(lh)	151	Limbic_TempPole(rh)	321
Cont_PFCl(lh)	291	Cont_Par(rh)	167
Default_Par(lh)	263	Cont_PFCl(rh)	543
Default_Temp(lh)	359	Default_Par(rh)	183
Default_PFC(lh)	771	Default_Temp(rh)	269
Default_PCC(lh)	281	Default_PFCv(rh)	60
		Default_PFCm(rh)	461
		Default_PCC(rh)	225

**Figure 1:**
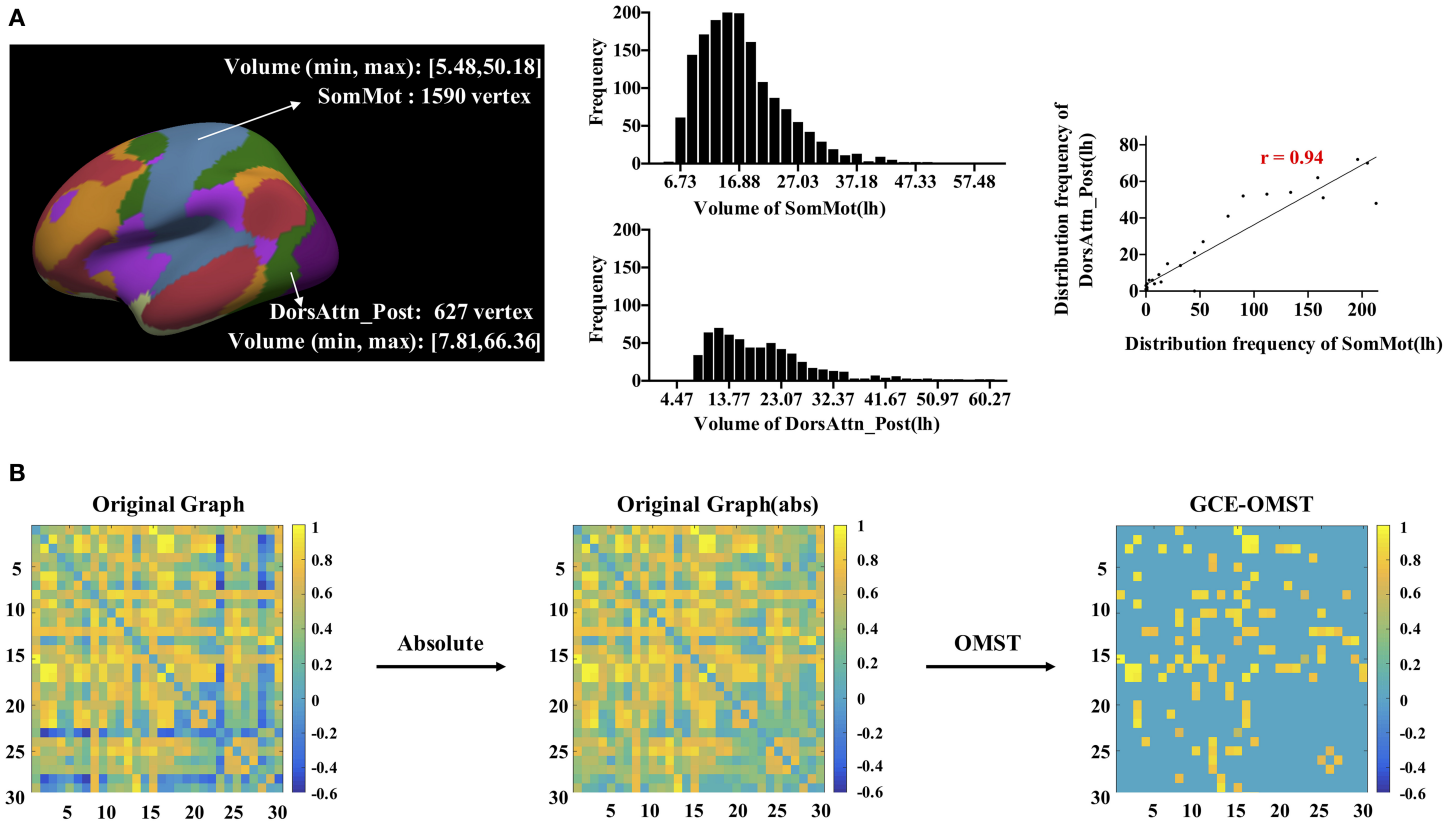
The workflow for the construction and thresholding of the morphological network for cortical volume. **(A)** For each pair of parcels, like the left SomMot and the left DorsAttn_Post, we uniformly divided their volumes into 30 bins (5.4–66.4). Each bin had a width of (66.4–5.4)/30 = 2.03, and the frequency distribution histograms were presented in the middle. By computing the Pearson's correlation of two sets of frequencies, we got the similarity of these two parcels. **(B)** showed the thresholding schemes of the network, and we first got absolute value of each connection in the network and then applied the data-driven thresholding scheme based on orthogonal minimal spanning trees (OMST).

We proposed a new method to construct a morphological network, including cortical volume, surface area, and cortical thickness. In a word, we estimated the distribution similarity of each morphological measurement for each pair of parcels (see Figure 1A, take cortical volume as an example). First, for each pair of parcels, we uniformly divided both of their volumes into 30 bins. Second, we computed the vertex frequency for each bin of the two parcels so that we got the frequency distribution histogram for each parcel. Third, the Pearson's correlation was calculated to estimate the volume distribution similarity, and we got a 32 × 32 morphological correlation matrix for each participant. The positive and negative connectivity, respectively, mean that the two brain regions have activation and inhibition effects. Significant inhibition effects should also be taken into account when calculating the entire network topology. Therefore, in this study, we considered the absolute values of connections to compute network efficiency. Considering orthogonal minimal spanning trees (OMST; Dimitriadis et al., 2017) as a threshold-free method to extract the strongest and the most important connections of a network, we used it to get an undirected weighted graph as shown in Figure 1B, and further, the network efficiency was computed based on the binary (unweighted) correlation matrix.

### Network Efficiency

We applied the graph theory (Achard et al., 2006) to compute global efficiency Eglob, nodal efficiency Enodal, and local efficiency Eloc (in more detail, we used the Brain Connectivity Toolbox, http://www.brain-connectivity-toolbox.net; Rubinov and Sporns, 2010).

In the graph theory, the global efficiency for a network G is defined as:

(1) $$\begin{array}{r} E_{glob}\left(G\right)=\frac{1}{N\left(N-1\right)}\sum_{i,j,i\neq j\in G}\frac{1}{L_{ij}} \end{array}$$

where *N* is the number of nodes and L_ij_ is the shortest path length between nodes *i* and node *j* in graph G (Latora and Marchiori, 2003). It was a global measure of the parallel information transfer ability of the whole network. The nodal efficiency of a node *i* is defined as:

(2) $$\begin{array}{r} E_{nodal}\left(i\right)=\frac{1}{N-1}\sum_{j,i\neq j\in G}\frac{1}{L_{ij}} \end{array}$$

where *N* and *L*_*ij*_ are the same as that in Equation (1), respectively the number of nodes and the shortest path length between nodes *i* and *j* in graph G (Latora and Marchiori, 2003). The nodal efficiency measures the importance of the node for the information transfer in the network. The local efficiency of a node *i* is defined as:

(3) $$\begin{array}{r} E_{local}\left(i\right)=E_{glob}\left(G_{i}\right) \end{array}$$

where G_i_ is a subgraph and is composed of the nodes that connected to node *i* (not including node *i*) directly and to interconnected edges. The local efficiency indicated how well the information was exchanged in the given subgraph.

### Statistical Analysis

To investigate whether the association between the brain network efficiency and intelligence was age dependent, we added an interactive term age × network efficiency in the general linear model that took age, morphological network efficiency (E, global efficiency, nodal efficiency, or local efficiency), sex, education, intracranial volume (ICV), and total cortical volume for volume network (or total surface area for area network or mean thickness for thickness network) as covariates. The detailed statistical model is shown in Equation (4).

(4) $$\begin{array}{l} beh=\alpha_{1}\times age+\alpha_{2}\times E+\alpha_{3}\times sex+\alpha_{4}\times edu+\alpha_{5}\\ \;\times ICV+\alpha_{6}\times morp_{total/mean}+\beta \times age\times E+\gamma \end{array}$$

False discovery rate (FDR, q < 0.05) correction for 32 parcels was used to control type 1 error over multiple tests. Also, the general linear model statistical analysis was performed using MATLAB scripts including regress.m and mafdr.m. Moreover, we further tested the reproducibility of the results using the leave-one-out (LOO) method. The LOO reproducibility was performed as follows: after one subject was randomly removed from the 65 subjects, we did the linear regression model on the remaining 64 subjects, which was repeated for 65 times (${\text{C}}_{65}^{1}$). The reproducibility was defined as the proportion of occurrences out of 65.

## Results

### Behavior Data

Table 3 illustrates detailed information about WAIS and SSEIS for the two groups, including their average, SD, maximum, and minimum. There were no significant differences between the two groups with respect to the total score and each dimension of IQ and SSEIS. Within the raw scores of 10 subsets in WAIS, only similarity subtests showed no significant differences between young adults and the middle-aged group; in other nine subsets, the middle-aged group always had a poorer performance than young adults possibly because of aging. However, raw scores of EI exhibited no significant differences between young adults and the middle-aged group.

**Table 3:** Descriptive statistics of Wechsler Adult Intelligence Scale and Schutte Self-Report Emotional Intelligence Scale test.

	**Young adults**		**Middle-aged group**	
	**Mean ± SD**	**Range**	**Mean ± SD**	**Range**
General ability index	121.06 ± 11.01	**98**−146	122.93 ± 13.07	102–144
Cognitive proficiency index	118.24 ± 11.09	92–142	119 ± 10.94	98–147
Verbal comprehension index	121.97 ± 10.42	93–145	122.57 ± 10.55	107–149
Perceptual reasoning index	115.18 ± 12.14	84–142	117.6 ± 16.45	94–144
Working memory index	113.53 ± 11.69	80–134	115.63 ± 13.44	89–148
Processing speed index	118.56 ± 13.54	92–145	117.67 ± 11.21	94–138
**FSIQ**	121.41 ± 10.77	97–142	122.93 ± 11.83	103–141
Emotion perception	46.53 ± 6.29	33–58	45.52 ± 5.7	30–56
Emotion management of the self	33.53 ± 2.94	28–38	32.65 ± 3.67	26–40
Emotion management of others	25.35 ± 3.2	17–30	25.29 ± 3.13	21–30
Emotion utilization	30.79 ± 3.84	21–35	29.03 ± 3.89	20–35
**EI**	136.21 ± 13.96	110–157	132.48 ± 13.57	105–161

*SD, standard deviation*.

### Inverted Age × Global Efficiency Interactions in Cognitive Intelligence and EI

**Figure 2:**
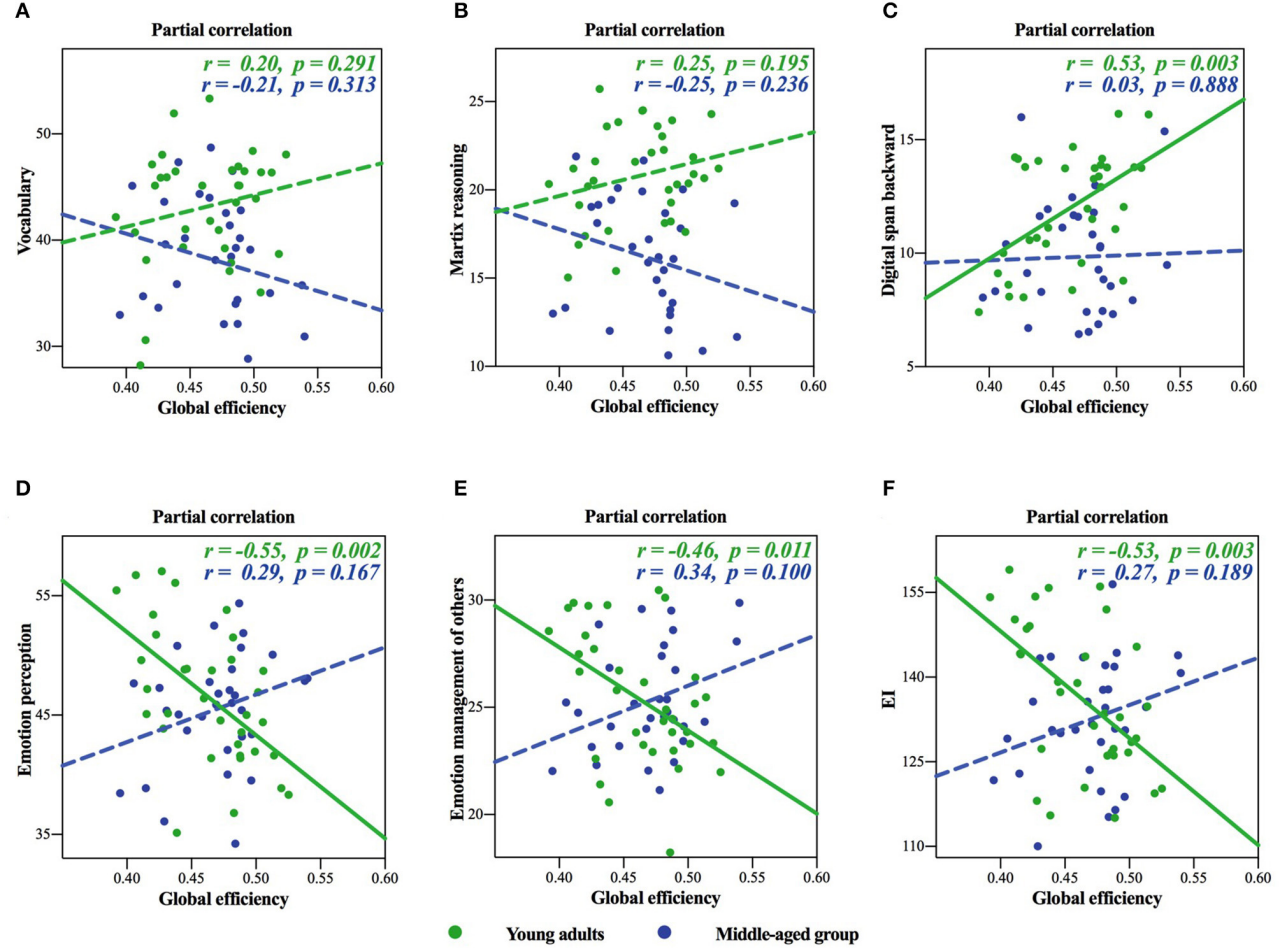
Inverted age × global efficiency interactions of the surface area network in cognitive intelligence and emotional intelligence (EI). **(A**–**C)** showed cognitive intelligence, and **(D**–**F)** showed EI. The solid lines represented the statistically significant subgroup correlations, whereas the dashed lines represented the subgroup correlations that did not pass the significance test or the false discovery rate (FDR) correction.

In the surface area network, we found significant negative age × global efficiency interactions in the raw scores of cognitive intelligence subtest (see Table 4 for details), while there was significant positive age × global efficiency interactions in EI (see Table 5 for details): these meant different age-dependent patterns for both cognitive intelligence and EI. For an intuitive illustration of the global efficiency as well as cognitive intelligence and EI, we also plotted scatters for each significant interaction in Figure 2. We plotted the partial correlations of the global efficiency with cognitive abilities for each age group. For young adults, the global efficiency predicted cognitive intelligence positively (Figures 2A–C), while for middle-aged group, the lower global efficiency was related to the higher cognitive intelligence (Figures 2D–F). Inversely, EI was negatively related to global efficiency in young adults but positively related to global efficiency in the middle-aged group.

**Table 4:** The significant age × network efficiency interactions in cognitive intelligence.

	**Morphological network**	**Reproducibility (%)**	**Significant interactions**		**Beta coefficient**	**Corrected *p*-value**
			**Parcel**	**Behaviors**		
Eglob	Area network	96.92	Global efficiency	Vocabulary	−0.27	0.019
		80.00	Global efficiency	Matrix reasoning	−0.19	0.039
		98.46	Global efficiency	Digital span backward	−0.25	0.021
Enodal	Volume network	89.23	Limbic_TempPole(rh)	Processing speed index	−0.38	0.031
	Area network	96.92	SalVentAttn_ParOper(lh)	General ability index	−0.38	0.016
		98.46	SalVentAttn_ParOper(lh)	Perceptual reasoning index	−0.40	0.015
		87.69	SalVentAttn_ParOper(lh)	FSIQ	−0.37	0.031
		100.00	SalVentAttn_ParOper(lh)	Matrix reasoning	−0.33	0.008
		69.23	Cont_Par(lh)	Vocabulary	−0.36	0.043
Eloc	Volume network	90.77	Default_PFCv(rh)	Digital span	0.37	0.027
		95.38	Default_PFCv(rh)	Digital span forward	0.39	0.020
	Area network	93.85	Cont_PFCl(rh)	Vocabulary	0.36	0.023

*Eglob, global efficiency; Enodal, nodal efficiency; Eloc, local efficiency; Beta coefficient, the standardized regression coefficient of age × efficiency in the general linear regression models; the corrected p-value, the significance for regression coefficient of age × efficiency (corrected by the false discovery rate [FDR] correction for 32 brain parcels); lh, left hemisphere; rh, right hemisphere; Limbic_TempPole, temporal pole; SalVentAttn_ParOper(lh), temporal-parietal junction (TPJ); Cont_Par(lh), inferior parietal sulcus (IPS); Default_PFCv, ventral medial prefrontal cortex (vmPFC); Cont_PFCl, anterior dorsal lateral prefrontal cortex (aDLPFC). We employed a leave-one-out process to assess the reproducibility of the significant interactions, which means that the data of 64 out of 65 subjects were randomly selected for the further analysis*.

**Table 5:** The significant age × network efficiency interactions in emotional intelligence.

	**Morphological network**	**Reproducibility (%)**	**Significant interactions**		**Beta coefficient**	**Corrected *p*-value**
			**Parcel**	**Behaviors**		
Eglob	Area network	100.00	Global efficiency	Emotion perception	0.31	0.013
		100.00	Global efficiency	Emotion management of others	0.33	0.012
		100.00	Global efficiency	EI	0.30	0.018
Enodal	Area network	67.69	SomMot(rh)	Emotion perception	0.38	0.040
		98.46	SomMot(rh)	Emotion management of the self	0.44	0.011
		55.38	SomMot(rh)	Emotion management of others	0.36	0.046
		100.00	SomMot(rh)	EI	0.43	0.019
		75.38	Limbic_TempPole(lh)	Emotion perception	0.41	0.040
		58.46	Limbic_TempPole(lh)	Emotion management of others	0.39	0.046
		95.38	Limbic_TempPole(lh)	EI	0.41	0.030
		80.00	Cont_Par(lh)	Emotion perception	0.42	0.040
		78.46	Cont_Par(lh)	Emotion management of others	0.43	0.038
		96.92	Cont_Par(lh)	EI	0.43	0.019
		52.31	Default_Par(lh)	Emotion management of others	0.34	0.046
		56.92	Default_PFC(lh)	Emotion management of others	0.36	0.046
		53.85	Default_PCC(lh)	Emotion management of others	0.34	0.046
		47.69	Default_PCC(rh)	Emotion management of others	0.34	0.046
Eloc	–					

*Eglob, global efficiency; Enodal, nodal efficiency; Eloc, local efficiency; Beta coefficient, the standardized regression coefficient of age × efficiency in the general linear regression models; corrected p-value, the significance for regression coefficient of age × efficiency (corrected by the false discovery rate [FDR] correction for 32 brain parcels); lh, left hemisphere; rh, right hemisphere; SomMot, sensory motor area (SMA); Limbic_TempPole, temporal pole; Cont_Par(lh), inferior parietal sulcus (IPS); Default_Par, lateral parietal cortex (LPC); Default_PFC, medial PFC (mPFC); Default_PCC, posterior cingulate cortex (PCC). We employed a leave-one-out process to assess the reproducibility of the significant interactions, which means that the data of 64 out of 65 subjects were randomly selected for the further analysis*.

### Inverted Age × Nodal Efficiency Interactions in Cognitive Intelligence and EI

In the volume network, we found significant negative age × nodal efficiency interactions in cognitive intelligence in the Limbic_TempPole(rh). In the area network, we found inverted age × nodal efficiency interactions in cognitive intelligence and EI: significant negative age × nodal efficiency interactions in the SalVentAttn_ParOper(lh) and Cont_Par(lh) (see Table 2 for detail), while significant positive age × nodal efficiency interactions in EI in the SomMot(rh), Limbic_TempPole(lh) Cont_Par(lh), Default_Par(lh), Default_PFC(lh), Default_PCC(lh), and Default_PCC(rh) (see Table 5 for detail). There was no significant age × nodal efficiency interaction in cognitive intelligence or EI in the cortical thickness network.

### Positive Age × Local Efficiency Interactions in Cognitive Intelligence

**Figure 3:**
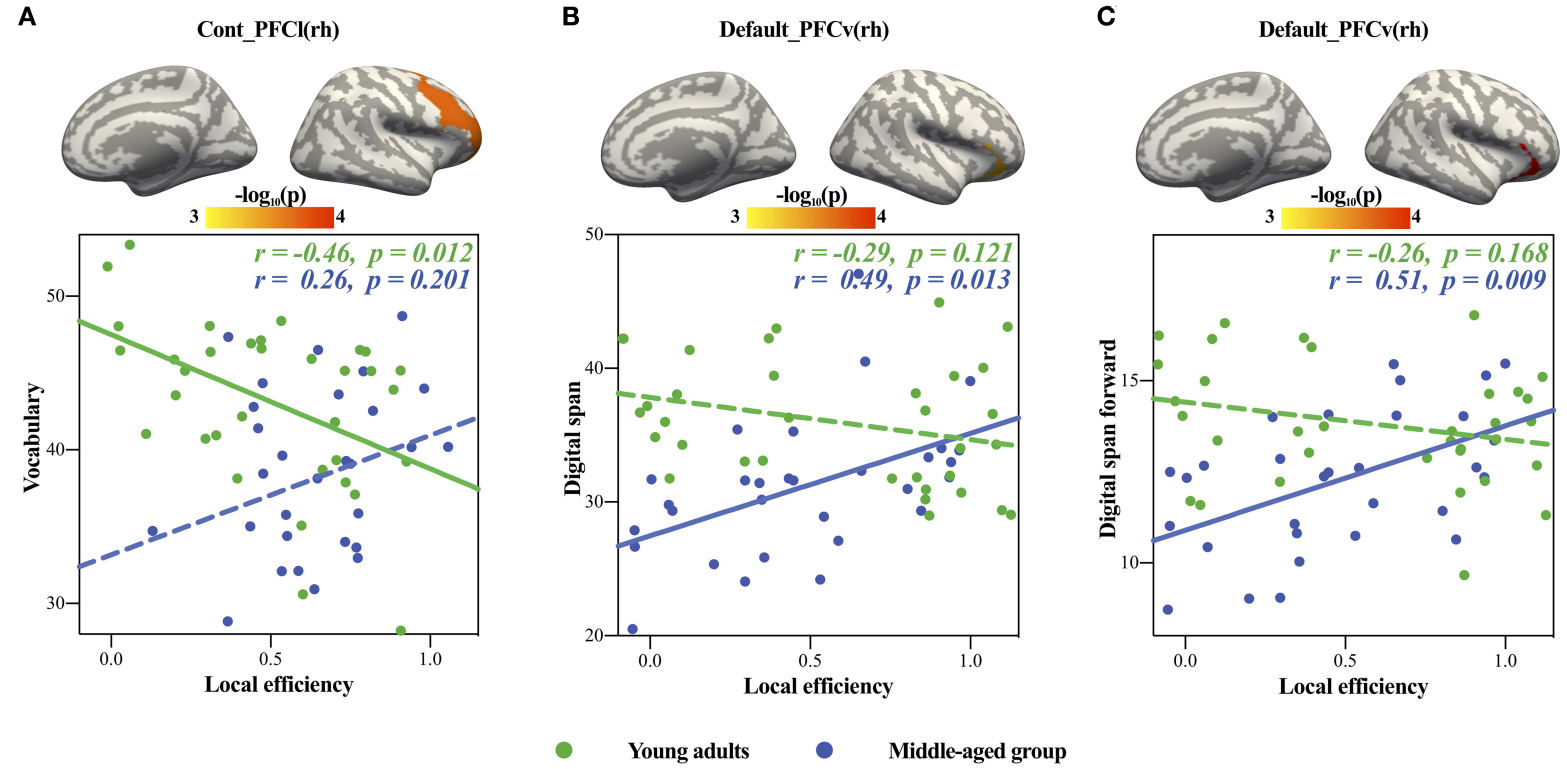
Significant age × local efficiency interactions in cognitive intelligence. The upper panels showed the locations of brain regions with significant age × local efficiency interactions, and the panels below showed scatters of each significant interaction. **(A,B)** showed volume network, and **(C)** showed area network. The solid lines represented the statistically significant subgroup correlations, whereas the dashed lines represented the subgroup correlations that did not pass the significance test or the FDR correction.

Only significant positive age × local efficiency interactions were found in cognitive intelligence in the Default_PFCv(rh) and Cont_PFCl(rh) (see Table 4 for detail). We also plotted scatters for the two different age groups. For young adults, we found negative correlations between digit span in the cognitive intelligence test and local efficiency in the Default_PFCv(rh) of the volume network (Figures 3A,B), as well as the negative correlation of vocabulary in cognitive intelligence test with local efficiency in the Cont_PFCl(rh) of area network (Figure 3C). The correlations above were reversed in the middle-aged group.

## Discussion

In this study, we found (1) associations between network efficiency and intelligence including cognitive intelligence and EI were age dependent; (2) the negative age × global efficiency interactions in cognitive intelligence implied stronger correlation of global efficiency with cognitive intelligence in young adults than the middle-aged group and emphasized the importance of parallel information transfer and integrated processing in young adults; and (3) on the other hand, the positive age × global efficiency interactions in EI implied stronger correlation of global efficiency with EI in the middle-aged group than young adults, and this may rely on increased connections between different networks such as sensory motor network, limbic network, frontoparietal network, and default mode network. In a summary, our findings demonstrated that global efficiency of cortical networks facilitated cognitive abilities for young adults; however, due to dynamic changes of between- and within-network connectivity, the correlation between global efficiency and cognitive intelligence decreased with aging, whereas for EI, the correlation between EI and global efficiency increased with age.

### Inverted Age × Global Efficiency Interactions in Cognitive Intelligence and EI

Cognitive intelligence and EI are two components of the human intelligence. Our results indicated that they had different developmental trajectories from young adults to the middle-aged group, which was consistent with previous studies, namely, cognitive intelligence decreased with advancing aging, but EI was relatively well-preserved across the two age groups (Salthouse, 2009, 2012; Mather, 2012, 2016). From the brain mechanism perspective, the neural underpinnings of intelligence also exhibited age-dependent patterns, more specifically, a positive correlation between cognitive intelligence and global efficiency, as well as nodal efficiency in young adults but inverted in the middle-aged group, and a positive correlation between EI and global efficiency, as well as nodal efficiency in the middle-aged group but inverted in young adults. Previous studies using structural and functional MRI data illustrated that the cognitive-related brain regions (mainly the frontoparietal network) showed age-related decrease in GM volume and within-network connectivity (Good et al., 2001; Tisserand et al., 2002; Fjell et al., 2009) and increased between-network connectivity (Avelar-Pereira et al., 2017); older adults showed less segregated network organization than young adults (Betzel et al., 2014; Cao et al., 2014). Also, the greater within-network connectivity (more segregated network organization) corresponded to the higher cognitive ability such as working memory (Damoiseaux et al., 2008), while the greater between-network connectivity was related to the poorer performance of cognitive test such as WAIS vocabulary test (Li et al., 2012). Combining the results of previous findings, our results further verified that the global efficiency as a measurement of the global information transfer ability became more vulnerable and the predictive value decreased in the middle-aged group compared to young adults (Ng et al., 2016; Tsvetanov et al., 2016; Petrican et al., 2017). However, there was no significant volume declines in emotional-related regions, mainly the emotion network (SEN, Seeley et al., 2007), the limbic network, and the paralimbic network, including the amygdala, anterior cingulate cortex, and subcortical areas (Salat et al., 2004; Grieve et al., 2005; Fjell et al., 2009; Lemaitre et al., 2012). The connectivity between the subcortical and paralimbic structures increased, implying improved emotion processing in the middle-aged group. Combining with the enhanced connectivity between the prefrontal cortex and the limbic areas (Kober et al., 2008; Nashiro et al., 2017), EI of middle-aged group may benefit more from higher global efficiency of the cortical networks (Smith et al., 2018b).

### Different Brain Mechanisms of Cognitive Intelligence in Young Adult and Middle-Aged Group

The human brain is composed of segregated brain networks and regions, which has independent functions and interrelate with each other to make sure the integration of information across different system (Watts and Strogatz, 1998). Previous studies emphasized the importance of parallel information transfer and integrated processing in cognitive abilities (Li et al., 2009; van den Heuvel et al., 2009). In the morphological network of cortical surface area, our findings showed that the higher global efficiency was associated with the higher scores in cognitive tests (digital span backward) in young adults. However, there was also a study that did not report any significant correlations between global efficiency and cognitive intelligence (Hilger et al., 2017), and a meta-analysis research concluded that there was no relationship between these two variables (Kruschwitz et al., 2018). The reason of this inconsistency may lie in different age distributions of participants. In this study, we found that the middle-aged group showed no relationship or even negative relationship between global efficiency and cognitive abilities. Cognitive intelligence declined when aging, probably due to the large-scale GM loss of frontal-parietal areas (Parkin, 1997; Good et al., 2001). Moreover, for middle-aged and elderly groups, previous studies showed that the less segregation among networks (i.e., less within-network connectivity; more between-network connectivity among ventral attention network, frontoparietal network, and default mode network) were related to the poorer cognitive performance (Betzel et al., 2014; Ng et al., 2016; Avelar-Pereira et al., 2017; DuPre and Spreng, 2017). In order to reduce the negative effect of volume loss and to reach the same cognitive level as young adults as possible, a successful cognitive aging is required with the preservation of interactions within and between brain networks (Dennis and Cabeza, 2011; Tsvetanov et al., 2016). On the other hand, connectivity density of the parietal area increased with age (Xu X. et al., 2015), and the most obvious change was that the regions specialized for a single cognitive function in young adults were coopted in support of multiple functions in older adults (the sensory system; Baltes and Lindenberger, 1997; the motor system; Carp et al., 2011; Du et al., 2016; the auditory system; Lalwani et al., 2019; and the visual system; De Sanctis et al., 2008); and these reflected a functional compensation of the human brain function while aging. Therefore, different brain mechanisms of cognitive intelligence from young adults to the middle-aged group in terms of global efficiency in the morphological network may result from less segregation between the brain regions and not a very clear division of the human brain functions (Xu X. et al., 2015; Muller et al., 2016).

Contrary to the global information transfer, we found significant positive age × local efficiency interactions in cognitive intelligence. The lower local efficiency in the ventral medial prefrontal cortex (vmPFC) of the volume network and the anterior dorsal lateral prefrontal cortex (aDLPFC) of the area network were associated with better performance in young adults. Local efficiency measured the ability of specialized information processing among topologically or anatomically nearby regions, which were simultaneously evoked during the localized functional activities (Bullmore and Sporns, 2009, 2012; Sporns, 2013). Meanwhile, the segregated information processing cannot fully cover the neural mechanism in all aspects of the brain functions. Considering that (1) prefrontal cortex was one of the brain regions for cognitive intelligence (Jung and Haier, 2007); (2) local efficiency decreased with age in 0–18 age groups (Gozdas et al., 2018); and (3) brain network changed from a localized organization to a distributed structure; thus, it was reasonable that cognitive intelligence was negatively associated with local efficiency in young adults in the vmPFC and the aDLPFC. For the middle-aged group, local efficiency was positively related to digit span in the cognitive intelligence test. Such local efficiency dependence may reflect a strategy to reduce the side effect of functional compassion and age-related neural dedifferentiation, and this was also verified by the evidence that the strong within-network connectivity had been proven to positively relate to cognitive performance (Damoiseaux et al., 2008; Mevel et al., 2013).

### Different Brain Mechanisms of EI in Young Adults and Middle-Aged Group

Early explorations of neurobiological substrates of EI mainly focused on the lesion data, and they proposed a somatic marker hypothesis (Damasio, 1994; Bar-On et al., 2003), which included the vmPFC), amygdala, and insular/somatosensory cortices. There were also some structural and functional MRI studies which verified the above results and illustrated the importance of limbic network, salient network, default mode network, and frontoparietal network (Smith et al., 2018a,b). Our findings on EI were mainly in the default mode network and were consistent with the previous studies.

The middle-aged group was characterized by the maturation of emotion control and regulation (Helson and Wink, 1987; Gruehn et al., 2007), and our results showed a positive correlation between the global efficiency in the area network and EI. The global efficiency of the cortical network corresponds to the global information processing among different brain regions, which has been proved to be crucial for emotion processing (Kinnison et al., 2012). Although studies found some discrete activation of the brain regions during different emotional experience (Kesler et al., 2001; Alkozei and Killgore, 2015; Quarto et al., 2016), a meta-analysis proved that the experience and perception of emotion emerge from a set of more basic psychological operational components (Lindquist et al., 2012), including the cooperation of the following components: information processing (the visual and sensory motor system, Ho and Lee, 2013; Satpute et al., 2015), core affect (limbic system; Heilman, 1997), perception (salient system, Barrett and Satpute, 2013), conceptualization (default mode system, Adhikari et al., 2010; Andrews-Hanna et al., 2014a,b), and executive attention (frontoparietal system, Sherman et al., 2014). Interestingly, in the area network of middle-aged group, we found positive correlations of EI and nodal efficiency in the sensory motor area (SMA: consists of primary motor area and primary somatosensory area, involving in the processing of somatosensory inputs in emotional experience), temporal pole, inferior parietal sulcus (IPS), and several default mode network regions, which were involved in the above conceptualization process of emotion. Besides, efficient communication between cortical areas was reported to be associated with higher emotional awareness (Smith et al., 2017).

However, for young adults, we found that the lower global efficiency of area network was related to the higher EI. Compared with the middle-aged group, young adults needed to pay more attention to external stimulation and had a higher activation of emotion experience (Castle et al., 2012; Spreng et al., 2017), which relied on the connectivity between cortical and subcortical areas and may reduce the importance of global efficiency in cortical network. The earlier-maturing visual and subcortical areas are in charge of primary functions including emotion perception as a type of stress response; the later-maturing cortical areas are in charge of higher-level and complex information processing and probably developed until the middle-aged group (Sowell et al., 2003; Schneider et al., 2004). With aging, the emotional information processing extended further into different aspects; hence, we observed different associations of global efficiency of area network with EI.

The cortical volume is a product of cortical thickness and surface area. The cortical thickness is associated with the number of neurons per column in the cerebral cortex, whereas the surface area is related to the number of column units (Rakic, 2009). Three measures have proved to be determined by different biological and neurodevelopmental factors, and they are differently associated with behaviors across the age span. Cox et al. (2018) found that the cortical volume and the surface area, but not cortical thickness, were associated with cognitive aging in older adults. In other words, the surface area has greater sensitivity to lifetime cognitive aging (Cox et al., 2016). Our results were in line with the findings showing that the network efficiency of the area network is more sensitive to the prediction of behaviors.

## Strengths and Limitations

The present study explored that the developmental brain mechanism of intelligence across a large age range, especially the differences between cognitive intelligence and EI, has been fully illustrated. Furthermore, we tried to explore the impact of interactions between networks of the brain regions on the individual differentiated behavior from the perspective of the brain networks, considering the dynamic changes of between- and within-network connectivity across life span (Chen et al., 2011; Chan et al., 2014).

Besides, there were some limitations: (1) considering that intensity inhomogeneity correction was essential for the preprocessing of structure MRI, in future studies, we might choose and apply more efficient correction methods; (2) the cross-section data may exist some problems to explore the developmental changes of neural mechanism; further studies could explore this change from a longitudinal dataset; (3) our subjects were not fully representative of the general population, which means that the subjects were likely more intelligent on average than the general population (mean IQ > 120). This study has a large proportion of people with higher levels of education and intelligence, which may, to some extent, limit the extent to which the results can be generalized and applied.

## Conclusion

Compared with the previous studies investigating the associations of network efficiency with cognitive intelligence and EI in adolescent or old people (Galvan et al., 2007; Saad et al., 2019), we recruited young adults and middle-aged people as subjects to explore the early aging process. More importantly, we proposed a new method to characterize the morphological network of the human brain, which reflected morphology similarity of brain regions and would add new insights on the studies of the human brain organization and neural dedifferentiation. Our research emphasized the developmental effects on the brain mechanisms of cognitive intelligence and EI from young adults to the middle-aged group and may promote mental health study of the middle-aged group in the future.

## Data Availability Statement

The datasets generated for this study are available on request to the corresponding author.

## Ethics Statement

The studies involving human participants were reviewed and approved by the institutional review board of Institute of Psychology Chinese Academy of Sciences. The patients/participants provided their written informed consent to participate in this study.

## Author Contributions

LJ and YM: conceptualization. LJ and CL: methodology. CL, KQ, and LJ: formal analysis and investigation. CL, LJ, and YM: writing and editing. All authors contributed to the article and approved the submitted version.

## Conflict of Interest

The authors declare that the research was conducted in the absence of any commercial or financial relationships that could be construed as a potential conflict of interest.
